# 2,9-Dimethyl-1,10-phenanthrolin-1-ium (6-carb­oxy-4-hy­droxy­pyridine-2-carboxyl­ato-κ^3^ *O* ^2^,*N*,*O* ^6^)(4-hy­droxy­pyridine-2,6-dicarboxyl­ato-κ^3^ *O* ^2^,*N*,*O* ^6^)nickelate(II) 2.35-hydrate: a proton-transfer compound

**DOI:** 10.1107/S1600536810031399

**Published:** 2010-08-11

**Authors:** Maryam Derakhshandeh, Zohreh Derikvand, Andya Nemati, Helen Stoeckli-Evans

**Affiliations:** a Department of Chemistry, Faculty of Science, Islamic Azad University, Mahshahr Branch, Mahshahr, Iran; b Department of Chemistry, Faculty of Sciences, Islamic Azad University, Khorramabad Branch, Khorramabad, Iran; c Iran Compiling Encyclopedia Foundation, Tajrish, Tehran, Iran; d Institute of Physics, University of Neuchâtel, rue Emile-Argand 11, CH-2009 Neuchâtel, Switzerland

## Abstract

The title proton-transfer compound, (C_14_H_13_N_2_)[Ni(C_7_H_3_NO_5_)(C_7_H_4_NO_5_)]·2.35H_2_O, consists of an [Ni(hypydc)(hypydcH)]^−^ anion, a dmpH^+^ cation and 2.35 uncoordinated water mol­ecules (where hypydcH_2_ = 4-hy­droxy­pyridine-2,6-dicarb­oxy­lic acid and dmp = 2,9-dimethyl-1,10-phenanthroline). The Ni^II^ atom is coordinated by two N atoms and four O atoms from the carboxyl­ate groups of the (hypydc)^2−^ and (hypydcH)^−^ ligands, forming a distorted octa­hedral environment. In the anion, the two pyridine rings are inclined to one another by 89.24 (10)°. In the crystal, cations are linked *via* O—H⋯O hydrogen bonds forming dimers, graph-set [*R*_2_^2^(16)], centered about inversion centers. These dimers are further linked by other cation O—H⋯O hydrogen bonds, graph-set [*R*_6_^6^(42)], forming a two-dimensional network in (011). Additional inter­molecular O—H⋯O, N—H⋯O, N—H⋯N, and weak C—H⋯O hydrogen bonds, and π–π inter­actions [shortest centroid–centroid distance = 3.5442 (14) Å], connect the two dimensional networks, forming a three-dimensional arrangement. The H atoms of one of the methyl groups are disordered over two sites with equal occupancy.

## Related literature

For literature on some first-row transition metal complexes of 4-hy­droxy­pyridine-2,6-dicarboxlic acid and various bases, see: Aghabozorg, Roshan *et al.* (2008 ▶); Aghabozorg, Saadaty *et al.* (2008 ▶); Aghabozorg, Motyeian *et al.* (2008 ▶); Ghadermazi *et al.* (2009 ▶); Rafizadeh *et al.* (2008 ▶); Ramos Silva *et al.* (2008 ▶). For details of graph-set analysis, see: Bernstein *et al.* (1995 ▶).
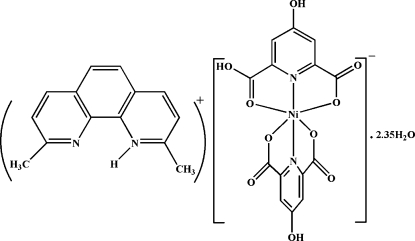


## Experimental

### 

#### Crystal data

(C_14_H_13_N_2_)[Ni(C_7_H_3_NO_5_)(C_7_H_4_NO_5_)]·2.35H_2_O
*M* *_r_* = 673.53
Monoclinic, 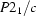

*a* = 11.1663 (8) Å
*b* = 9.7296 (9) Å
*c* = 25.698 (2) Å
β = 94.330 (9)°
*V* = 2784.0 (4) Å^3^
*Z* = 4
Mo *K*α radiation
μ = 0.77 mm^−1^
*T* = 223 K
0.34 × 0.30 × 0.23 mm

#### Data collection

Stoe IPDS diffractometer
Absorption correction: multi-scan (*MULscanABS* in *PLATON*; Spek, 2009 ▶) *T*_min_ = 0.820, *T*_max_ = 0.839
21508 measured reflections
5442 independent reflections
3321 reflections with *I* > 2σ(*I*)
*R*_int_ = 0.049

#### Refinement

*R*[*F* ^2^ > 2σ(*F* ^2^)] = 0.032
*wR*(*F* ^2^) = 0.071
*S* = 0.82
5442 reflections
439 parameters
6 restraints
H atoms treated by a mixture of independent and constrained refinement
Δρ_max_ = 0.29 e Å^−3^
Δρ_min_ = −0.49 e Å^−3^

### 

Data collection: *EXPOSE* (Stoe & Cie, 2000 ▶); cell refinement: *CELL* (Stoe & Cie, 2000 ▶); data reduction: *INTEGRATE* (Stoe & Cie, 2000 ▶); program(s) used to solve structure: *SHELXS97* (Sheldrick, 2008 ▶); program(s) used to refine structure: *SHELXL97* (Sheldrick, 2008 ▶); molecular graphics: *PLATON* (Spek, 2009 ▶) and *Mercury* (Macrae *et al.*, 2006 ▶); software used to prepare material for publication: *SHELXL97* and *PLATON* (Spek, 2009 ▶).

## Supplementary Material

Crystal structure: contains datablocks I, global. DOI: 10.1107/S1600536810031399/lh5110sup1.cif

Structure factors: contains datablocks I. DOI: 10.1107/S1600536810031399/lh5110Isup2.hkl

Additional supplementary materials:  crystallographic information; 3D view; checkCIF report

## Figures and Tables

**Table 1:** Hydrogen-bond geometry (Å, °) *Cg*1 is the centroid of the N1,C1–C5.

*D*—H⋯*A*	*D*—H	H⋯*A*	*D*⋯*A*	*D*—H⋯*A*
O1*W*—H1*WA*⋯O9^i^	0.82 (2)	2.03 (3)	2.832 (3)	166 (4)
O1*W*—H1*WB*⋯O4^ii^	0.84 (3)	2.15 (3)	2.942 (3)	158 (3)
O2—H2*O*⋯O7^iii^	0.83	1.65	2.399 (2)	148
N3—H3⋯O1*W*	0.87	2.01	2.845 (3)	161
N3—H3⋯N4	0.87	2.38	2.728 (3)	105
O2*W*—H2*WA*⋯O8^iv^	0.84 (3)	1.99 (3)	2.785 (3)	156 (3)
O2*W*—H2*WA*⋯O9^iv^	0.84 (3)	2.55 (3)	3.267 (3)	144 (3)
O2*W*—H2*WB*⋯O4	0.81 (2)	2.28 (2)	3.094 (3)	176 (5)
O5—H5*O*⋯O2*W*^v^	0.83	1.75	2.570 (3)	169
O10—H10*O*⋯O3^vi^	0.83	1.81	2.597 (2)	158
C27—H27*C*⋯O3^vii^	0.97	2.55	3.480 (4)	161
C22—H22⋯*Cg*1^iv^	0.94	2.76	3.635 (3)	155

Symmetry codes: (i) 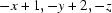; (ii) 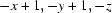; (iii) 
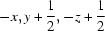
; (iv) 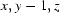; (v) 
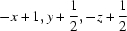
; (vi) 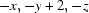; (vii) 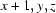.

**Fig. 1.**
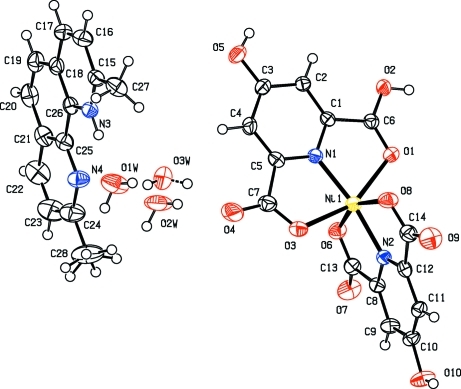
The molecular structure of the title compound with the displacement ellipsoids drawn at the 50% probability level.

**Fig. 2.**
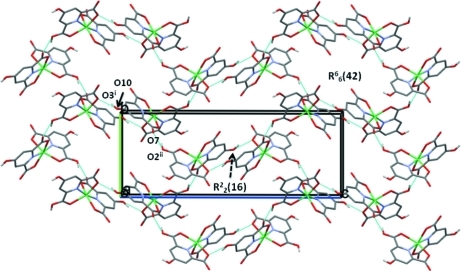
A view along the a-axis of the O-H···O hydrogen bonded two-dimensional network in (011), showing the formation of the graph-sets [R22(16)] and [R66(42)]. H-atoms not involved in hydrogen bonding (cyan lines) have been omitted for clarity; symmetry codes: (i) -x, -y+2, -z; (ii) -x, y-0.5, -z+0.5.

**Fig. 3.**
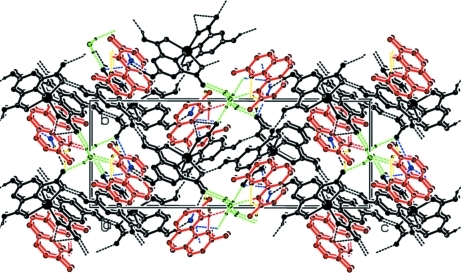
A view of along the a-axis of the crystal packing in the title compound. The Nickel complex is shown in black, the dmpH+ cation in red, and the water molecules are in blue, green and yellow; the various hydrogen bonds are shown as dashed lines - see Table 1 for details.

## supplementary crystallographic information

### Comment

The crystal structures of a number of first-row proton transfer complexes, where 4-hydroxypyridine-2,6-dicarboxlic acid (hypydcH_2_) is the proton donor, have been reported previously (Ramos Silva *et al.*, 2008; Aghabozorg, Saadaty *et al.*, 2008; Aghabozorg, Roshan *et al.*, 2008; Rafizadeh *et al.*, 2008; Aghabozorg, Motyeian *et al.*, 2008; Ghadermazi *et al.* 2009). Herein, we present the crystal structure of the title complex, prepared by the reaction of nickel(II) nitrate with the same proton donnor (hypydcH_2_) and the proton acceptor 2,9-dimethyl-1,10-phenanthroline (dmp).

The asymmetric unit of the title compound consists of one [Ni(hypydc)(hypydcH)]^-^ anion, one 2,9-dimethyl-1,10-phenanthrolinium cation (dmpH^+^) and 2.35 uncoordinated water molecules (Fig. 1). A carboxylic acid proton has been transferred to an N atom of 2,9-dimethyl-1,10-phenanthroline. In the anions, the Ni^II^ atom is six-coordinated by two N atoms (N1 and N2) that occupy the axial positions, and four O atoms, (O1, O3, O6 and O8) from the carboxylate groups of the (hypydc)^2-^and (hypydcH)^-^ ligands, in the equitorial plane, so forming a distorted octahedral geometry. The (hypydc)^2-^and (hypydcH)^-^ ligands are orthogonal; the two pyridine ring mean planes being inclined to one another by 89.24 (10)°. This geometry is simlar to that in the proton transfer nickel(II) complex Bis(guanidinium)bis(4-hydroxypyridine-2,6-dicarboxylato- *κ^3^O^2^,N,O^6^*)nickelate(II) dihydrate (Aghabozorg, Motyeian *et al.*, 2008). In the dmpH^+^ cation there is a short N-H···N interaction as a result of the inherant planarity of the system (Table 1).

In the crystal the cations are linked via O–H···O hydrogen bonds to form dimers - graph-set [R^2^_2_(16)] - centered about inversion centers (Bernstein *et al.*, 1995; Table 1, Fig 2). These dimers are further link by other cation O-H···O hydrogen bonds - graph-set [R^6^_6_(42)] - to form a two-dimensional network in (011). Additional intermolecular O–H···O, N–H···O, N–H···N, and weak C–H···O hydrogen bonds connect these two dimensional networks to form a three-dimensional arrangeemt (Fig. 3).

Another feature of the crystal structure of the title compound is the presence of π–π stacking interactions (Table 2). The shortest π–π distance is 3.5442 (14) Å involving pyridine ring (N2/C8-C12) of the (hypydc)^2-^ anion and the central aromatic ring of the dmpH^+^ cation (C18-C21,C25-C26).

Footnote for Table 1: Cg1 is the centroid of ring N1,C1-C5.

### Experimental

An aqueous solution of nickel(II) nitrate hexahydrate (0.5 mmol, 90 mg) in distilled water (5 ml) was added to an aqueous solution of 4-hydroxypyridine-2,6-dicarboxylic acid(1 mmol, 183 mg) in distilled water (20 ml) and 2,9-dimethyl-1,10-phenanthroline (1 mmol, 208 mg) in methanol (5 ml) under stirring at 333K in a 1:2:2 molar ratio for lh. Blue block-shaped crystals were obtained by slow evaporation of the solvent at the room temperature.

### Refinement

The water molecule O3W was only partially occupied and was refined with an occupancy of 0.35. Methyl group C28 was treated as an idealized disordered methyl group with two positions rotated from each other by 60°; each H-atom occupancy was set to 0.5. The water H-atoms were refined with distance restraints of O-H = 0.84 (2) Å and U_iso_(H) = 1.5U_eq_(O). The OH, NH and C-bound H-atoms were included in calculated positions and treated as riding on their parent atom: O-H = 0.83 Å, N-H = 0.87 Å, and C-H = 0.94 and 0.97 Å, for CH and CH_3_, respectively, with U_iso_(H) = k × U_eq_(O, N, or C), where k = 1.5 for CH_3_ H-atoms and 1.2 for all other H-atoms.

### Crystal data

**Table:**

(C_14_H_13_N_2_)[Ni(C_7_H_3_NO_5_)(C_7_H_4_NO_5_)]·2.35H_2_O	*F*(000) = 1390
*M**_r_* = 673.53	*D*_x_ = 1.607 Mg m^−^^3^
Monoclinic, *P*2_1_/*c*	Mo *K*α radiation, λ = 0.71073 Å
Hall symbol: -P 2ybc	Cell parameters from 8000 reflections
*a* = 11.1663 (8) Å	θ = 2.2–26.1°
*b* = 9.7296 (9) Å	µ = 0.77 mm^−^^1^
*c* = 25.698 (2) Å	*T* = 223 K
β = 94.330 (9)°	Block, pale-blue
*V* = 2784.0 (4) Å^3^	0.34 × 0.30 × 0.23 mm
*Z* = 4	

### Data collection

**Table:**

Stoe IPDS diffractometer	5442 independent reflections
Radiation source: fine-focus sealed tube	3321 reflections with *I* > 2σ(*I*)
graphite	*R*_int_ = 0.049
φ rotation scans	θ_max_ = 26.1°, θ_min_ = 2.2°
Absorption correction: multi-scan (MULscanABS in *PLATON*; Spek, 2009)	*h* = −13→12
*T*_min_ = 0.820, *T*_max_ = 0.839	*k* = −12→11
21508 measured reflections	*l* = −31→31

### Refinement

**Table:**

Refinement on *F*^2^	Secondary atom site location: difference Fourier map
Least-squares matrix: full	Hydrogen site location: inferred from neighbouring sites
*R*[*F*^2^ > 2σ(*F*^2^)] = 0.032	H atoms treated by a mixture of independent and constrained refinement
*wR*(*F*^2^) = 0.071	*w* = 1/[σ^2^(*F*_o_^2^) + (0.0379*P*)^2^] where *P* = (*F*_o_^2^ + 2*F*_c_^2^)/3
*S* = 0.82	(Δ/σ)_max_ = 0.001
5442 reflections	Δρ_max_ = 0.29 e Å^−^^3^
439 parameters	Δρ_min_ = −0.49 e Å^−^^3^
6 restraints	Extinction correction: *SHELXL97* (Sheldrick, 2008), Fc^*^=kFc[1+0.001xFc^2^λ^3^/sin(2θ)]^-1/4^
Primary atom site location: structure-invariant direct methods	Extinction coefficient: 0.0014 (3)

### Special details

**Table:**

Geometry. Bond distances, angles etc. have been calculated using the rounded fractional coordinates. All su's are estimated from the variances of the (full) variance-covariance matrix. The cell esds are taken into account in the estimation of distances, angles and torsion angles
Refinement. The water H-atoms were refined with distance restraints of O-H = 0.84 (2) Å and U_iso_(H) = 1.5U_eq_(O). The OH, NH and C-bound H-atoms were included in calculated positions and treated as riding on their parent atom: O-H = 0.83 Å, N-H = 0.87 Å, C-H = 0.94 and 0.97 Å, for CH and CH_3_, respectively, with U_iso_(H) = k × U_eq_(O, N, or C), where k = 1.5 for CH_3_ H-atoms and 1.2 for all other H-atoms. The water molecule O3W was only partially occupied and was refined with an occupancy of 0.35. Methyl group C28 was treated as an idealized disordered methyl group with two positions rotated from each other by 60°; each H-atom occupancy was set to 0.5.

### Fractional atomic coordinates and isotropic or equivalent isotropic displacement parameters (Å^2^)

**Table:**

	*x*	*y*	*z*	*U*_iso_*/*U*_eq_	Occ. (<1)
Ni1	0.14739 (3)	0.96793 (3)	0.15130 (1)	0.0260 (1)	
O1	0.15046 (15)	1.07916 (16)	0.22542 (6)	0.0298 (5)	
O2	0.25528 (15)	1.07951 (16)	0.30382 (6)	0.0290 (5)	
O3	0.21156 (15)	0.82051 (17)	0.09837 (6)	0.0313 (6)	
O4	0.34942 (19)	0.6562 (2)	0.09309 (8)	0.0662 (9)	
O5	0.58914 (17)	0.7378 (2)	0.27078 (7)	0.0453 (7)	
O6	0.01044 (16)	0.82870 (16)	0.17517 (6)	0.0308 (5)	
O7	−0.18300 (17)	0.77766 (19)	0.15269 (7)	0.0428 (7)	
O8	0.21250 (15)	1.14097 (17)	0.11129 (6)	0.0340 (6)	
O9	0.15781 (17)	1.28829 (18)	0.04666 (7)	0.0472 (7)	
O10	−0.25820 (15)	1.06177 (19)	−0.01149 (6)	0.0405 (7)	
N1	0.28647 (17)	0.88779 (18)	0.19159 (7)	0.0232 (6)	
N2	0.00840 (16)	1.02066 (19)	0.10486 (6)	0.0234 (6)	
C1	0.3184 (2)	0.9328 (2)	0.23970 (8)	0.0226 (7)	
C2	0.4179 (2)	0.8853 (2)	0.26876 (8)	0.0251 (7)	
C3	0.4886 (2)	0.7856 (3)	0.24664 (9)	0.0295 (8)	
C4	0.4526 (2)	0.7357 (2)	0.19695 (9)	0.0305 (8)	
C5	0.3517 (2)	0.7890 (2)	0.17069 (8)	0.0276 (8)	
C6	0.2335 (2)	1.0398 (2)	0.25655 (8)	0.0240 (7)	
C7	0.3021 (2)	0.7496 (3)	0.11634 (9)	0.0333 (9)	
C8	−0.0908 (2)	0.9446 (2)	0.10399 (8)	0.0239 (7)	
C9	−0.1816 (2)	0.9572 (3)	0.06563 (8)	0.0282 (7)	
C10	−0.1691 (2)	1.0544 (2)	0.02645 (8)	0.0277 (8)	
C11	−0.0673 (2)	1.1354 (2)	0.02800 (8)	0.0254 (7)	
C12	0.0207 (2)	1.1152 (2)	0.06783 (8)	0.0248 (7)	
C13	−0.0863 (2)	0.8416 (2)	0.14803 (9)	0.0286 (8)	
C14	0.1396 (2)	1.1890 (2)	0.07497 (9)	0.0315 (8)	
N3	0.85534 (18)	0.40284 (19)	0.10413 (7)	0.0285 (6)	
N4	0.64764 (19)	0.2613 (2)	0.08013 (8)	0.0378 (8)	
C15	0.9561 (2)	0.4758 (3)	0.11221 (9)	0.0297 (7)	
C16	1.0299 (2)	0.4506 (3)	0.15731 (9)	0.0378 (9)	
C17	1.0007 (2)	0.3517 (3)	0.19177 (9)	0.0361 (9)	
C18	0.8949 (2)	0.2752 (2)	0.18279 (9)	0.0305 (8)	
C19	0.8601 (3)	0.1697 (3)	0.21716 (10)	0.0380 (9)	
C20	0.7587 (3)	0.0981 (3)	0.20579 (10)	0.0404 (10)	
C21	0.6824 (2)	0.1255 (3)	0.15974 (10)	0.0362 (9)	
C22	0.5769 (3)	0.0533 (3)	0.14537 (12)	0.0522 (11)	
C23	0.5108 (3)	0.0846 (3)	0.10047 (12)	0.0572 (11)	
C24	0.5487 (3)	0.1909 (3)	0.06806 (11)	0.0481 (10)	
C25	0.7134 (2)	0.2290 (3)	0.12506 (9)	0.0319 (8)	
C26	0.8214 (2)	0.3034 (2)	0.13757 (9)	0.0277 (8)	
C27	0.9852 (3)	0.5789 (3)	0.07223 (10)	0.0409 (9)	
C28	0.4771 (3)	0.2259 (4)	0.01801 (12)	0.0692 (14)	
O1W	0.7549 (2)	0.4759 (2)	0.00287 (7)	0.0628 (9)	
O2W	0.3478 (2)	0.3691 (2)	0.14479 (9)	0.0670 (9)	
O3W	0.5288 (6)	0.4962 (6)	0.1014 (2)	0.059 (2)	0.350
H2	0.43790	0.91880	0.30260	0.0300*	
H2O	0.21100	1.14540	0.30970	0.0430*	
H4	0.49690	0.66640	0.18170	0.0370*	
H5O	0.60140	0.77650	0.29950	0.0680*	
H9	−0.25060	0.90200	0.06560	0.0340*	
H10O	−0.23970	1.11730	−0.03410	0.0610*	
H11	−0.05830	1.20280	0.00240	0.0310*	
H3	0.80880	0.41940	0.07610	0.0340*	
H16	1.10050	0.50230	0.16410	0.0450*	
H17	1.05200	0.33470	0.22180	0.0430*	
H19	0.90830	0.15030	0.24790	0.0460*	
H20	0.73740	0.02830	0.22860	0.0480*	
H22	0.55160	−0.01730	0.16690	0.0630*	
H23	0.43990	0.03570	0.09090	0.0690*	
H27A	0.91230	0.62570	0.05920	0.0610*	
H27B	1.02040	0.53280	0.04360	0.0610*	
H27C	1.04180	0.64540	0.08780	0.0610*	
H28A	0.48890	0.32200	0.00980	0.1040*	0.500
H28B	0.39260	0.20920	0.02190	0.1040*	0.500
H28C	0.50360	0.16920	−0.01000	0.1040*	0.500
H28D	0.43450	0.14490	0.00470	0.1040*	0.500
H28E	0.53080	0.25780	−0.00740	0.1040*	0.500
H28F	0.41980	0.29770	0.02440	0.1040*	0.500
H1WA	0.769 (4)	0.551 (2)	−0.0102 (14)	0.0940*	
H1WB	0.715 (3)	0.426 (3)	−0.0187 (12)	0.0940*	
H2WA	0.307 (3)	0.312 (3)	0.1262 (13)	0.1000*	
H2WB	0.352 (4)	0.445 (2)	0.1319 (14)	0.1000*	
H3WA	0.506 (9)	0.576 (5)	0.091 (4)	0.0880*	0.350
H3WB	0.568 (8)	0.470 (11)	0.077 (3)	0.0880*	0.350

### Atomic displacement parameters (Å^2^)

**Table:**

	*U*^11^	*U*^22^	*U*^33^	*U*^12^	*U*^13^	*U*^23^
Ni1	0.0257 (2)	0.0274 (2)	0.0242 (2)	−0.0010 (2)	−0.0024 (1)	0.0008 (1)
O1	0.0285 (10)	0.0309 (9)	0.0290 (8)	0.0058 (7)	−0.0042 (8)	−0.0019 (7)
O2	0.0354 (10)	0.0282 (9)	0.0232 (8)	0.0086 (7)	0.0003 (7)	−0.0042 (7)
O3	0.0323 (11)	0.0367 (10)	0.0243 (8)	−0.0016 (8)	−0.0021 (8)	−0.0020 (7)
O4	0.0577 (15)	0.0876 (17)	0.0509 (12)	0.0291 (13)	−0.0117 (11)	−0.0451 (12)
O5	0.0437 (13)	0.0535 (12)	0.0366 (10)	0.0256 (10)	−0.0112 (9)	−0.0100 (9)
O6	0.0339 (11)	0.0323 (9)	0.0257 (8)	0.0008 (8)	−0.0002 (8)	0.0056 (7)
O7	0.0367 (12)	0.0463 (11)	0.0451 (11)	−0.0130 (9)	0.0020 (9)	0.0193 (9)
O8	0.0284 (10)	0.0358 (10)	0.0366 (10)	−0.0092 (8)	−0.0044 (8)	0.0017 (8)
O9	0.0497 (13)	0.0363 (11)	0.0551 (12)	−0.0168 (9)	0.0013 (10)	0.0150 (9)
O10	0.0315 (11)	0.0624 (14)	0.0265 (9)	−0.0060 (9)	−0.0059 (8)	0.0124 (8)
N1	0.0231 (11)	0.0243 (10)	0.0220 (10)	−0.0004 (8)	0.0006 (8)	−0.0015 (8)
N2	0.0249 (11)	0.0219 (9)	0.0231 (9)	−0.0007 (9)	0.0001 (8)	0.0001 (8)
C1	0.0248 (13)	0.0226 (12)	0.0206 (11)	−0.0019 (9)	0.0030 (10)	−0.0012 (9)
C2	0.0294 (14)	0.0252 (12)	0.0204 (11)	0.0012 (10)	−0.0007 (10)	0.0002 (9)
C3	0.0295 (15)	0.0299 (12)	0.0284 (12)	0.0061 (11)	−0.0021 (11)	0.0021 (10)
C4	0.0321 (15)	0.0297 (13)	0.0299 (13)	0.0089 (11)	0.0032 (11)	−0.0043 (10)
C5	0.0316 (15)	0.0271 (13)	0.0240 (12)	−0.0025 (11)	0.0021 (11)	−0.0039 (10)
C6	0.0256 (13)	0.0215 (11)	0.0247 (11)	−0.0025 (11)	0.0012 (10)	0.0008 (10)
C7	0.0313 (16)	0.0401 (16)	0.0282 (13)	0.0000 (12)	0.0007 (11)	−0.0057 (12)
C8	0.0230 (13)	0.0253 (13)	0.0235 (11)	−0.0025 (10)	0.0029 (10)	−0.0006 (9)
C9	0.0220 (13)	0.0364 (14)	0.0260 (11)	−0.0068 (11)	0.0015 (10)	0.0017 (11)
C10	0.0261 (14)	0.0361 (15)	0.0206 (11)	0.0042 (11)	0.0001 (10)	0.0015 (10)
C11	0.0277 (14)	0.0269 (13)	0.0218 (11)	0.0022 (11)	0.0029 (10)	0.0040 (9)
C12	0.0287 (14)	0.0193 (12)	0.0269 (12)	−0.0021 (10)	0.0050 (10)	−0.0005 (9)
C13	0.0339 (16)	0.0272 (13)	0.0247 (12)	−0.0012 (12)	0.0027 (12)	0.0021 (10)
C14	0.0311 (16)	0.0301 (14)	0.0334 (14)	−0.0032 (11)	0.0023 (12)	−0.0019 (11)
N3	0.0318 (12)	0.0292 (11)	0.0243 (10)	0.0022 (9)	0.0007 (9)	−0.0026 (8)
N4	0.0284 (13)	0.0515 (14)	0.0333 (12)	−0.0057 (11)	0.0021 (10)	−0.0028 (10)
C15	0.0296 (14)	0.0297 (12)	0.0299 (12)	−0.0017 (12)	0.0029 (10)	−0.0064 (11)
C16	0.0349 (16)	0.0407 (16)	0.0373 (14)	−0.0067 (13)	−0.0009 (12)	−0.0061 (12)
C17	0.0386 (17)	0.0383 (15)	0.0301 (13)	0.0061 (13)	−0.0061 (12)	−0.0049 (11)
C18	0.0362 (16)	0.0290 (13)	0.0266 (12)	0.0072 (11)	0.0051 (11)	−0.0043 (10)
C19	0.0487 (19)	0.0346 (15)	0.0314 (14)	0.0103 (13)	0.0069 (13)	0.0016 (11)
C20	0.056 (2)	0.0307 (14)	0.0364 (15)	0.0029 (14)	0.0161 (14)	0.0036 (12)
C21	0.0389 (17)	0.0351 (14)	0.0362 (14)	−0.0027 (12)	0.0137 (13)	−0.0034 (11)
C22	0.051 (2)	0.056 (2)	0.0519 (18)	−0.0163 (16)	0.0190 (15)	−0.0012 (15)
C23	0.0418 (19)	0.074 (2)	0.0570 (19)	−0.0245 (16)	0.0112 (16)	−0.0073 (16)
C24	0.0316 (17)	0.069 (2)	0.0441 (16)	−0.0098 (15)	0.0051 (13)	−0.0071 (14)
C25	0.0298 (15)	0.0364 (14)	0.0304 (13)	−0.0007 (11)	0.0081 (11)	−0.0047 (11)
C26	0.0304 (15)	0.0268 (13)	0.0267 (12)	0.0023 (11)	0.0066 (11)	−0.0038 (10)
C27	0.0492 (18)	0.0384 (15)	0.0354 (14)	−0.0116 (13)	0.0053 (13)	−0.0033 (11)
C28	0.037 (2)	0.112 (3)	0.057 (2)	−0.0174 (19)	−0.0064 (16)	−0.0023 (19)
O1W	0.0982 (19)	0.0473 (13)	0.0392 (11)	−0.0271 (13)	−0.0194 (11)	0.0131 (10)
O2W	0.0778 (18)	0.0594 (16)	0.0572 (14)	−0.0216 (13)	−0.0377 (12)	0.0033 (11)
O3W	0.061 (4)	0.048 (4)	0.066 (4)	0.019 (3)	−0.007 (3)	−0.007 (3)

### Geometric parameters (Å, °)

**Table:**

Ni1—O1	2.1887 (16)	C8—C9	1.365 (3)
Ni1—O3	2.1373 (17)	C8—C13	1.510 (3)
Ni1—O6	2.1660 (17)	C9—C10	1.396 (3)
Ni1—O8	2.1291 (17)	C10—C11	1.381 (3)
Ni1—N1	1.9619 (19)	C11—C12	1.378 (3)
Ni1—N2	1.9536 (17)	C12—C14	1.508 (3)
O1—C6	1.239 (3)	C2—H2	0.9400
O2—C6	1.280 (3)	C4—H4	0.9400
O3—C7	1.281 (3)	C9—H9	0.9400
O4—C7	1.229 (3)	C11—H11	0.9400
O5—C3	1.325 (3)	C15—C16	1.392 (3)
O6—C13	1.247 (3)	C15—C27	1.489 (4)
O7—C13	1.260 (3)	C16—C17	1.364 (4)
O8—C14	1.280 (3)	C17—C18	1.401 (3)
O9—C14	1.235 (3)	C18—C19	1.427 (4)
O10—C10	1.341 (3)	C18—C26	1.398 (3)
O2—H2O	0.8300	C19—C20	1.343 (5)
O5—H5O	0.8300	C20—C21	1.430 (4)
O10—H10O	0.8300	C21—C22	1.397 (4)
O1W—H1WA	0.82 (2)	C21—C25	1.405 (4)
O1W—H1WB	0.84 (3)	C22—C23	1.357 (4)
O2W—H2WB	0.81 (2)	C23—C24	1.413 (4)
O2W—H2WA	0.84 (3)	C24—C28	1.501 (4)
O3W—H3WB	0.83 (8)	C25—C26	1.422 (3)
O3W—H3WA	0.85 (6)	C16—H16	0.9400
N1—C5	1.342 (3)	C17—H17	0.9400
N1—C1	1.335 (3)	C19—H19	0.9400
N2—C12	1.338 (3)	C20—H20	0.9400
N2—C8	1.331 (3)	C22—H22	0.9400
N3—C15	1.333 (3)	C23—H23	0.9400
N3—C26	1.367 (3)	C27—H27B	0.9700
N4—C24	1.317 (4)	C27—H27A	0.9700
N4—C25	1.358 (3)	C27—H27C	0.9700
N3—H3	0.8700	C28—H28F	0.9700
C1—C2	1.371 (3)	C28—H28A	0.9700
C1—C6	1.494 (3)	C28—H28B	0.9700
C2—C3	1.398 (3)	C28—H28C	0.9700
C3—C4	1.397 (3)	C28—H28D	0.9700
C4—C5	1.371 (3)	C28—H28E	0.9700
C5—C7	1.513 (3)		
			
O1—Ni1—O3	154.38 (6)	C3—C2—H2	121.00
O1—Ni1—O6	91.59 (6)	C3—C4—H4	120.00
O1—Ni1—O8	92.62 (6)	C5—C4—H4	120.00
O1—Ni1—N1	77.14 (7)	C8—C9—H9	121.00
O1—Ni1—N2	111.18 (7)	C10—C9—H9	121.00
O3—Ni1—O6	92.20 (6)	C12—C11—H11	121.00
O3—Ni1—O8	94.60 (6)	C10—C11—H11	121.00
O3—Ni1—N1	77.24 (7)	C16—C15—C27	123.1 (2)
O3—Ni1—N2	94.39 (7)	N3—C15—C16	118.4 (2)
O6—Ni1—O8	154.88 (7)	N3—C15—C27	118.5 (2)
O6—Ni1—N1	98.61 (7)	C15—C16—C17	120.6 (2)
O6—Ni1—N2	77.99 (7)	C16—C17—C18	120.5 (2)
O8—Ni1—N1	106.47 (7)	C17—C18—C19	123.1 (2)
O8—Ni1—N2	77.40 (7)	C19—C18—C26	119.0 (2)
N1—Ni1—N2	170.92 (8)	C17—C18—C26	117.9 (2)
Ni1—O1—C6	111.77 (14)	C18—C19—C20	120.2 (2)
Ni1—O3—C7	115.18 (15)	C19—C20—C21	121.6 (3)
Ni1—O6—C13	112.27 (14)	C20—C21—C22	124.3 (3)
Ni1—O8—C14	114.80 (14)	C22—C21—C25	115.8 (2)
C6—O2—H2O	109.00	C20—C21—C25	119.9 (2)
C3—O5—H5O	109.00	C21—C22—C23	120.4 (3)
C10—O10—H10O	109.00	C22—C23—C24	119.9 (3)
H1WA—O1W—H1WB	111 (3)	N4—C24—C28	117.8 (3)
H2WA—O2W—H2WB	114 (3)	N4—C24—C23	121.7 (3)
H3WA—O3W—H3WB	102 (10)	C23—C24—C28	120.5 (3)
Ni1—N1—C1	120.26 (15)	N4—C25—C21	124.2 (2)
C1—N1—C5	119.38 (19)	C21—C25—C26	117.8 (2)
Ni1—N1—C5	120.35 (14)	N4—C25—C26	118.0 (2)
C8—N2—C12	120.02 (18)	C18—C26—C25	121.5 (2)
Ni1—N2—C8	119.08 (14)	N3—C26—C18	119.2 (2)
Ni1—N2—C12	119.71 (15)	N3—C26—C25	119.3 (2)
C15—N3—C26	123.4 (2)	C15—C16—H16	120.00
C24—N4—C25	118.1 (2)	C17—C16—H16	120.00
C15—N3—H3	118.00	C18—C17—H17	120.00
C26—N3—H3	118.00	C16—C17—H17	120.00
N1—C1—C6	111.48 (18)	C18—C19—H19	120.00
C2—C1—C6	125.65 (19)	C20—C19—H19	120.00
N1—C1—C2	122.87 (19)	C19—C20—H20	119.00
C1—C2—C3	118.13 (19)	C21—C20—H20	119.00
O5—C3—C4	118.8 (2)	C23—C22—H22	120.00
C2—C3—C4	118.7 (2)	C21—C22—H22	120.00
O5—C3—C2	122.5 (2)	C22—C23—H23	120.00
C3—C4—C5	119.2 (2)	C24—C23—H23	120.00
C4—C5—C7	126.1 (2)	C15—C27—H27B	110.00
N1—C5—C4	121.64 (19)	H27A—C27—H27C	109.00
N1—C5—C7	112.27 (19)	C15—C27—H27C	110.00
O1—C6—O2	126.8 (2)	H27A—C27—H27B	109.00
O1—C6—C1	119.26 (18)	C15—C27—H27A	109.00
O2—C6—C1	113.95 (18)	H27B—C27—H27C	109.00
O3—C7—C5	114.9 (2)	C24—C28—H28C	110.00
O4—C7—C5	119.7 (2)	C24—C28—H28E	109.00
O3—C7—O4	125.4 (2)	C24—C28—H28F	110.00
N2—C8—C9	122.3 (2)	C24—C28—H28D	109.00
C9—C8—C13	125.6 (2)	C24—C28—H28A	109.00
N2—C8—C13	112.08 (18)	C24—C28—H28B	109.00
C8—C9—C10	118.2 (2)	H28A—C28—H28E	56.00
C9—C10—C11	119.5 (2)	H28A—C28—H28F	56.00
O10—C10—C9	116.83 (19)	H28B—C28—H28C	110.00
O10—C10—C11	123.65 (19)	H28B—C28—H28D	56.00
C10—C11—C12	118.62 (19)	H28B—C28—H28E	141.00
N2—C12—C14	112.17 (18)	H28B—C28—H28F	56.00
N2—C12—C11	121.4 (2)	H28C—C28—H28D	56.00
C11—C12—C14	126.44 (19)	H28C—C28—H28E	56.00
O7—C13—C8	114.8 (2)	H28C—C28—H28F	141.00
O6—C13—O7	127.6 (2)	H28D—C28—H28E	110.00
O6—C13—C8	117.68 (19)	H28D—C28—H28F	109.00
O8—C14—C12	114.87 (18)	H28E—C28—H28F	109.00
O8—C14—O9	126.2 (2)	H28A—C28—H28B	109.00
O9—C14—C12	118.9 (2)	H28A—C28—H28C	109.00
C1—C2—H2	121.00	H28A—C28—H28D	141.00
			
O3—Ni1—O1—C6	−0.8 (2)	C24—N4—C25—C21	−0.3 (4)
O6—Ni1—O1—C6	97.65 (15)	C25—N4—C24—C23	0.0 (4)
O8—Ni1—O1—C6	−107.12 (15)	C25—N4—C24—C28	−179.3 (3)
N1—Ni1—O1—C6	−0.82 (15)	C24—N4—C25—C26	178.9 (2)
N2—Ni1—O1—C6	175.38 (14)	N1—C1—C2—C3	0.2 (3)
O1—Ni1—O3—C7	2.0 (3)	N1—C1—C6—O2	175.37 (18)
O6—Ni1—O3—C7	−96.33 (17)	C6—C1—C2—C3	−179.3 (2)
O8—Ni1—O3—C7	107.87 (17)	N1—C1—C6—O1	−3.4 (3)
N1—Ni1—O3—C7	2.00 (17)	C2—C1—C6—O1	176.1 (2)
N2—Ni1—O3—C7	−174.44 (17)	C2—C1—C6—O2	−5.2 (3)
O1—Ni1—O6—C13	114.42 (15)	C1—C2—C3—C4	−2.2 (3)
O3—Ni1—O6—C13	−90.93 (15)	C1—C2—C3—O5	177.0 (2)
O8—Ni1—O6—C13	14.8 (2)	C2—C3—C4—C5	2.2 (3)
N1—Ni1—O6—C13	−168.35 (15)	O5—C3—C4—C5	−177.1 (2)
N2—Ni1—O6—C13	3.09 (15)	C3—C4—C5—C7	178.3 (2)
O1—Ni1—O8—C14	−116.33 (15)	C3—C4—C5—N1	−0.1 (3)
O3—Ni1—O8—C14	88.27 (15)	N1—C5—C7—O3	3.5 (3)
O6—Ni1—O8—C14	−16.9 (2)	N1—C5—C7—O4	−176.9 (2)
N1—Ni1—O8—C14	166.30 (15)	C4—C5—C7—O4	4.5 (4)
N2—Ni1—O8—C14	−5.21 (15)	C4—C5—C7—O3	−175.1 (2)
O1—Ni1—N1—C1	−1.16 (16)	N2—C8—C9—C10	−0.5 (3)
O1—Ni1—N1—C5	−179.94 (17)	C13—C8—C9—C10	−177.3 (2)
O3—Ni1—N1—C1	178.86 (18)	N2—C8—C13—O6	−8.0 (3)
O3—Ni1—N1—C5	0.09 (16)	N2—C8—C13—O7	172.29 (19)
O6—Ni1—N1—C1	−90.83 (17)	C9—C8—C13—O6	169.1 (2)
O6—Ni1—N1—C5	90.40 (16)	C9—C8—C13—O7	−10.6 (3)
O8—Ni1—N1—C1	87.78 (17)	C8—C9—C10—O10	178.2 (2)
O8—Ni1—N1—C5	−90.99 (17)	C8—C9—C10—C11	−0.9 (3)
O1—Ni1—N2—C8	−95.09 (16)	C9—C10—C11—C12	1.6 (3)
O1—Ni1—N2—C12	97.40 (16)	O10—C10—C11—C12	−177.5 (2)
O3—Ni1—N2—C8	83.25 (16)	C10—C11—C12—C14	177.4 (2)
O3—Ni1—N2—C12	−84.27 (16)	C10—C11—C12—N2	−0.8 (3)
O6—Ni1—N2—C8	−8.07 (15)	N2—C12—C14—O8	6.5 (3)
O6—Ni1—N2—C12	−175.58 (17)	C11—C12—C14—O8	−171.8 (2)
O8—Ni1—N2—C8	176.99 (16)	C11—C12—C14—O9	8.8 (3)
O8—Ni1—N2—C12	9.48 (15)	N2—C12—C14—O9	−172.8 (2)
Ni1—O1—C6—O2	−176.12 (18)	N3—C15—C16—C17	1.3 (4)
Ni1—O1—C6—C1	2.5 (2)	C27—C15—C16—C17	−177.8 (3)
Ni1—O3—C7—O4	176.9 (2)	C15—C16—C17—C18	−1.0 (4)
Ni1—O3—C7—C5	−3.5 (3)	C16—C17—C18—C19	179.5 (2)
Ni1—O6—C13—O7	−178.64 (19)	C16—C17—C18—C26	0.2 (4)
Ni1—O6—C13—C8	1.7 (2)	C17—C18—C19—C20	−178.6 (3)
Ni1—O8—C14—O9	−179.95 (19)	C26—C18—C19—C20	0.6 (4)
Ni1—O8—C14—C12	0.8 (2)	C17—C18—C26—N3	0.3 (3)
Ni1—N1—C1—C2	−176.87 (16)	C17—C18—C26—C25	179.0 (2)
Ni1—N1—C1—C6	2.6 (2)	C19—C18—C26—N3	−179.0 (2)
C5—N1—C1—C2	1.9 (3)	C19—C18—C26—C25	−0.3 (3)
C5—N1—C1—C6	−178.60 (18)	C18—C19—C20—C21	−0.6 (4)
Ni1—N1—C5—C4	176.87 (16)	C19—C20—C21—C22	179.0 (3)
Ni1—N1—C5—C7	−1.8 (2)	C19—C20—C21—C25	0.3 (4)
C1—N1—C5—C4	−1.9 (3)	C20—C21—C22—C23	−178.9 (3)
C1—N1—C5—C7	179.42 (19)	C25—C21—C22—C23	−0.2 (4)
Ni1—N2—C8—C9	−166.20 (18)	C20—C21—C25—N4	179.2 (2)
Ni1—N2—C8—C13	11.0 (2)	C20—C21—C25—C26	0.0 (4)
C12—N2—C8—C9	1.3 (3)	C22—C21—C25—N4	0.4 (4)
C12—N2—C8—C13	178.47 (18)	C22—C21—C25—C26	−178.8 (2)
Ni1—N2—C12—C11	166.80 (16)	C21—C22—C23—C24	−0.1 (5)
Ni1—N2—C12—C14	−11.7 (2)	C22—C23—C24—N4	0.2 (5)
C8—N2—C12—C11	−0.6 (3)	C22—C23—C24—C28	179.5 (3)
C8—N2—C12—C14	−179.07 (18)	N4—C25—C26—N3	−0.5 (3)
C15—N3—C26—C18	0.0 (3)	N4—C25—C26—C18	−179.2 (2)
C26—N3—C15—C16	−0.8 (4)	C21—C25—C26—N3	178.7 (2)
C26—N3—C15—C27	178.4 (2)	C21—C25—C26—C18	0.0 (4)
C15—N3—C26—C25	−178.7 (2)		

### Hydrogen-bond geometry (Å, °)

**Table:**

Cg1 is the centroid of the N1,C1–C5.

**Table:**

*D*—H···*A*	*D*—H	H···*A*	*D*···*A*	*D*—H···*A*
O1W—H1WA···O9^i^	0.82 (2)	2.03 (3)	2.832 (3)	166 (4)
O1W—H1WB···O4^ii^	0.84 (3)	2.15 (3)	2.942 (3)	158 (3)
O2—H2O···O7^iii^	0.83	1.65	2.399 (2)	148
N3—H3···O1W	0.87	2.01	2.845 (3)	161
N3—H3···N4	0.87	2.38	2.728 (3)	105
O2W—H2WA···O8^iv^	0.84 (3)	1.99 (3)	2.785 (3)	156 (3)
O2W—H2WA···O9^iv^	0.84 (3)	2.55 (3)	3.267 (3)	144 (3)
O2W—H2WB···O4	0.81 (2)	2.28 (2)	3.094 (3)	176 (5)
O5—H5O···O2W^v^	0.83	1.75	2.570 (3)	169
O10—H10O···O3^vi^	0.83	1.81	2.597 (2)	158
C27—H27C···O3^vii^	0.97	2.55	3.480 (4)	161
C22—H22···Cg1^iv^	0.94	2.76	3.635 (3)	155

Symmetry codes: (i) −*x*+1, −*y*+2, −*z*; (ii) −*x*+1, −*y*+1, −*z*; (iii) −*x*, *y*+1/2, −*z*+1/2; (iv) *x*, *y*−1, *z*; (v) −*x*+1, *y*+1/2, −*z*+1/2; (vi) −*x*, −*y*+2, −*z*; (vii) *x*+1, *y*, *z*.

### Table 2 π···π interactions [Å] Cg1 is the centroid of ring N1,C1-C5; Cg2 is the centroid of ring N2,C8-C12; Cg3 is the centroid of ring N3,C15-C18,C26; Cg4 is the centroid of ring N4,C21-C25; Cg5 is the centroid of ring C18-C19,C25,C26; Symmetry codes : (i) -x+1, y+0.5, -z+0.5; (ii) x-1, y+1, z

**Table:**

CgI	CgJ	Centroid-to-Centroid
Cg1	Cg5^i^	3.7818 (14)
Cg2	Cg3^ii^	3.9022 (13)
Cg2	Cg4^ii^	3.8946 (15)
Cg2	Cg5^ii^	3.5442 (14)
